# Dimethyl 6-bromo-2-methyl-1,2-di­hydro­quinoline-2,4-dicarboxyl­ate

**DOI:** 10.1107/S1600536812005600

**Published:** 2012-02-17

**Authors:** Zeynep Gültekin, Michael Bolte, Tuncer Hökelek

**Affiliations:** a Department of Chemistry, Çankırı Karatekin University, TR-18100, Çankırı, Turkey; b Institut für Anorganische Chemie, J. W. Goethe-Universität Frankfurt, Max-von-Laue-Strasse 7, D-60438 Frankfurt/Main, Germany; c Department of Physics, Hacettepe University, 06800 Beytepe, Ankara, Turkey

## Abstract

In the title compound, C_14_H_14_BrNO_4_, the dihydro­pyridine ring adopts a screw-boat conformation. In the crystal, pairs of N—H⋯O hydrogen bonds link the mol­ecules into inversion *R*_2_^2^(10) dimers.

## Related literature

For the synthesis of 1,2-dihydro­quinolines, see: Hu *et al.* (2011 ▶); Yadav *et al.* (2007 ▶, 2008 ▶); Waldmann *et al.* (2008 ▶); Zhang & Ji (2011 ▶). For the biological activity of dihydro­quinolines, see: Craig & Pearson (1971 ▶); Muren & Weissman (1971 ▶); Hamann *et al.* (1998 ▶); He *et al.* (2003 ▶); LaMontagne *et al.* (1989 ▶). For related structures, see: Gültekin *et al.* (2010 ▶, 2011**a* ▶,b* ▶, 2012 ▶). For hydrogen-bond motifs, see: Bernstein *et al.* (1995 ▶). For ring-puckering parameters, see: Cremer & Pople (1975 ▶).
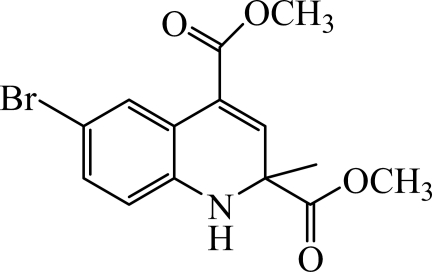


## Experimental

### 

#### Crystal data

C_14_H_14_BrNO_4_
*M* *_r_* = 340.16
Triclinic, 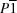

*a* = 7.8273 (9) Å
*b* = 10.4827 (11) Å
*c* = 10.5029 (12) Å
α = 115.837 (9)°
β = 105.655 (9)°
γ = 96.889 (8)°
*V* = 718.25 (17) Å^3^
*Z* = 2
Mo *K*α radiation
μ = 2.87 mm^−1^
*T* = 173 K
0.33 × 0.32 × 0.22 mm

#### Data collection

Stoe IPDS II two-circle diffractometer
Absorption correction: multi-scan (*MULABS*; Spek, 2009 ▶; Blessing, 1995 ▶) *T*_min_ = 0.451, *T*_max_ = 0.571
7423 measured reflections
2692 independent reflections
2267 reflections with *I* > 2σ(*I*)
*R*_int_ = 0.045

#### Refinement

*R*[*F* ^2^ > 2σ(*F* ^2^)] = 0.053
*wR*(*F* ^2^) = 0.153
*S* = 1.21
2692 reflections
189 parameters
1 restraint
H atoms treated by a mixture of independent and constrained refinement
Δρ_max_ = 0.96 e Å^−3^
Δρ_min_ = −0.46 e Å^−3^

### 

Data collection: *X-AREA* (Stoe & Cie, 2001 ▶); cell refinement: *X-AREA*; data reduction: *X-RED32* (Stoe & Cie, 2001 ▶); program(s) used to solve structure: *SHELXS97* (Sheldrick, 2008 ▶); program(s) used to refine structure: *SHELXL97* (Sheldrick, 2008 ▶); molecular graphics: *ORTEP-3 for Windows* (Farrugia, 1997 ▶); software used to prepare material for publication: *WinGX* (Farrugia, 1999 ▶) and *PLATON* (Spek, 2009 ▶).

## Supplementary Material

Crystal structure: contains datablock(s) I, global. DOI: 10.1107/S1600536812005600/xu5465sup1.cif

Structure factors: contains datablock(s) I. DOI: 10.1107/S1600536812005600/xu5465Isup2.hkl

Supplementary material file. DOI: 10.1107/S1600536812005600/xu5465Isup3.cml

Additional supplementary materials:  crystallographic information; 3D view; checkCIF report

## Figures and Tables

**Table 1:** Hydrogen-bond geometry (Å, °)

*D*—H⋯*A*	*D*—H	H⋯*A*	*D*⋯*A*	*D*—H⋯*A*
N1—H1⋯O1^i^	0.90 (8)	2.12 (8)	3.013 (6)	176 (7)

Symmetry code: (i) 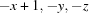.

**Fig. 1.**
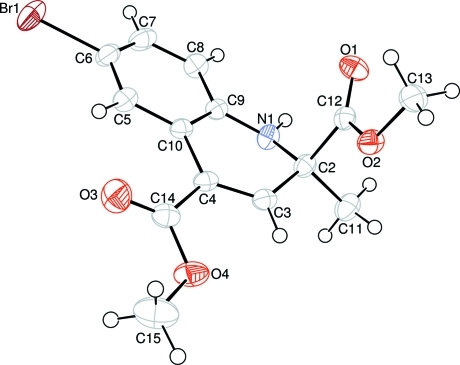
The molecular structure of the title compound with the atom-numbering scheme. Displacement ellipsoids are drawn at the 50% probability level.

**Fig. 2.**
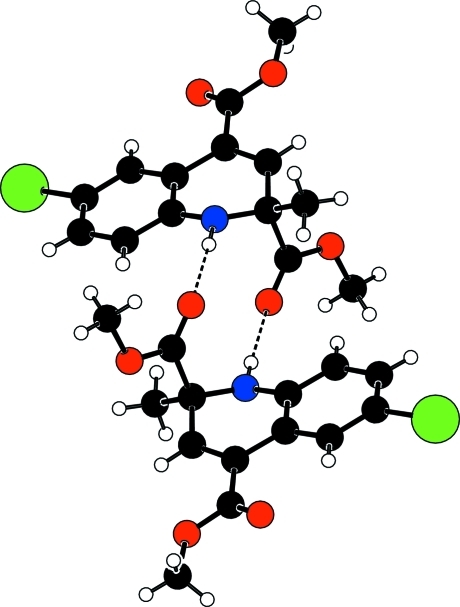
A partial packing diagram. Hydrogen bonds are shown as dashed lines.

## supplementary crystallographic information

### Comment

1,2-Dihydroquinoline derivatives have been considerably important for the preparation of biologically important compounds (Craig & Pearson, 1971; Muren & Weissman, 1971). Many methods have been reported in the literature for the preparation of 1,2-dihydroquinolines (Yadav *et al.*, 2007, 2008). The most convenient method is the condensation of aromatic amines with ketones using a catalytic amount of a Lewis acid or Brønsted acid (Hu *et al.*, 2011; Waldmann *et al.*, 2008; Zhang & Ji, 2011). Dihydroquinolines are also powerful intermediates for the preparation of quinolines and many quinolines display biological effects (Hamann *et al.*, 1998; LaMontagne *et al.*, 1989; He *et al.*, 2003).

The structures of some 1,2-dihydroquinoline derivatives, C_16_H_19_NO_4_ (Gültekin *et al.*, 2010), C_14_H_15_NO_4_ (Gültekin *et al.*, 2011*a*), C_17_H_21_NO_7_ (Gültekin *et al.*, 2011*b*) and C_16_H_17_NO_5_ (Gültekin *et al.*, 2012) have also been determined.

In the title compound, (I), (Fig. 1), the ring A (C2–C4/C9/C10/N1) is not planar, but adopting a screw-boat confromation with puckering parameters (Cremer & Pople, 1975) Q_T_ = 0.339 (5)Å, φ = -162.6(1.5)° and θ = 129.2(1.1)°.

In the crystal structure, intermolecular N—H···O hydrogen bonds (Table 1) link the molecules into centrosymmetric R_2_^2^(10) dimers (Bernstein *et al.*, 1995) (Fig. 2).

### Experimental

The title compound was synthesized by the literature method (Waldmann *et al.*, 2008). p-bromo aniline (100 mg, 1 eq) was dissolved in acetonitrile (1.5 ml), and then Bi(OTf)_3_ (5 mol%, 0.05 eq) and methyl pyruvate (2.2 eq) were added to the mixture. The mixture was heated by microwave irradiation for 7 h until the starting material was completely consumed as monitored by TLC. The resultant residue was directly purified by flash chromatography on silica (EtOAc:Cylohexane 1:2). Recrystallization over pentane and ethyl acetate (70:30) gave a yellow crystalline solid (yield: 81%), R_f_ 0.5 (2:1 Cyclohexane/EtOAc) m.p. 379–381 K.

### Refinement

Amino H atom was located in a difference map and refined isotropically, the C-bound H atoms were positioned geometrically with C—H = 0.93 and 0.96 Å, and constrained to ride on their parent atoms, with *U*_iso_(H) = k × *U*_eq_(C), where k = 1.2 for aromatic and k = 1.5 for methyl H atoms, respectively.

### Crystal data

**Table:**

C_14_H_14_BrNO_4_	*Z* = 2
*M**_r_* = 340.16	*F*(000) = 344
Triclinic, *P*1	*D*_x_ = 1.573 Mg m^−^^3^
Hall symbol: -P 1	Mo *K*α radiation, λ = 0.71073 Å
*a* = 7.8273 (9) Å	Cell parameters from 6851 reflections
*b* = 10.4827 (11) Å	θ = 3.5–25.9°
*c* = 10.5029 (12) Å	µ = 2.87 mm^−^^1^
α = 115.837 (9)°	*T* = 173 K
β = 105.655 (9)°	Block, yellow
γ = 96.889 (8)°	0.33 × 0.32 × 0.22 mm
*V* = 718.25 (17) Å^3^	

### Data collection

**Table:**

Stoe IPDS II two-circle diffractometer	2692 independent reflections
Radiation source: fine-focus sealed tube	2267 reflections with *I* > 2σ(*I*)
Graphite monochromator	*R*_int_ = 0.045
ω scans	θ_max_ = 25.6°, θ_min_ = 3.5°
Absorption correction: multi-scan (*MULABS*; Spek, 2009; Blessing, 1995)	*h* = −9→9
*T*_min_ = 0.451, *T*_max_ = 0.571	*k* = −12→12
7423 measured reflections	*l* = −12→12

### Refinement

**Table:**

Refinement on *F*^2^	Secondary atom site location: difference Fourier map
Least-squares matrix: full	Hydrogen site location: inferred from neighbouring sites
*R*[*F*^2^ > 2σ(*F*^2^)] = 0.053	H atoms treated by a mixture of independent and constrained refinement
*wR*(*F*^2^) = 0.153	*w* = 1/[σ^2^(*F*_o_^2^) + (0.0648*P*)^2^ + 1.4243*P*] where *P* = (*F*_o_^2^ + 2*F*_c_^2^)/3
*S* = 1.21	(Δ/σ)_max_ < 0.001
2692 reflections	Δρ_max_ = 0.96 e Å^−^^3^
189 parameters	Δρ_min_ = −0.46 e Å^−^^3^
1 restraint	Extinction correction: *SHELXL97* (Sheldrick, 2008), Fc^*^=kFc[1+0.001xFc^2^λ^3^/sin(2θ)]^-1/4^
Primary atom site location: structure-invariant direct methods	Extinction coefficient: 0.038 (5)

### Special details

**Table:**

Geometry. All esds (except the esd in the dihedral angle between two l.s. planes) are estimated using the full covariance matrix. The cell esds are taken into account individually in the estimation of esds in distances, angles and torsion angles; correlations between esds in cell parameters are only used when they are defined by crystal symmetry. An approximate (isotropic) treatment of cell esds is used for estimating esds involving l.s. planes.
Refinement. Refinement of F^2^ against ALL reflections. The weighted R-factor wR and goodness of fit S are based on F^2^, conventional R-factors R are based on F, with F set to zero for negative F^2^. The threshold expression of F^2^ > 2sigma(F^2^) is used only for calculating R-factors(gt) etc. and is not relevant to the choice of reflections for refinement. R-factors based on F^2^ are statistically about twice as large as those based on F, and R- factors based on ALL data will be even larger.

### Fractional atomic coordinates and isotropic or equivalent isotropic displacement parameters (Å^2^)

**Table:**

	*x*	*y*	*z*	*U*_iso_*/*U*_eq_	
Br1	0.98733 (9)	0.36331 (7)	0.80285 (6)	0.0521 (3)	
O1	0.3080 (4)	0.0758 (4)	0.0556 (4)	0.0340 (8)	
O2	0.2146 (4)	0.2722 (4)	0.0660 (4)	0.0309 (7)	
O3	0.7138 (6)	0.6908 (4)	0.6183 (4)	0.0453 (9)	
O4	0.6368 (5)	0.7295 (4)	0.4208 (4)	0.0400 (8)	
N1	0.6748 (5)	0.2278 (4)	0.1501 (4)	0.0280 (8)	
H1	0.678 (13)	0.138 (5)	0.085 (9)	0.11 (3)*	
C2	0.5298 (6)	0.2890 (5)	0.1017 (5)	0.0262 (9)	
C3	0.5671 (6)	0.4450 (5)	0.2227 (5)	0.0256 (9)	
H3	0.5245	0.5116	0.1950	0.031*	
C4	0.6599 (6)	0.4919 (5)	0.3699 (5)	0.0249 (9)	
C5	0.8118 (6)	0.4208 (5)	0.5664 (5)	0.0308 (10)	
H5	0.8093	0.5073	0.6451	0.037*	
C6	0.8886 (6)	0.3213 (6)	0.5976 (5)	0.0326 (10)	
C7	0.8965 (7)	0.1930 (6)	0.4853 (6)	0.0368 (11)	
H7	0.9516	0.1288	0.5096	0.044*	
C8	0.8212 (6)	0.1596 (5)	0.3345 (5)	0.0305 (10)	
H8	0.8243	0.0720	0.2577	0.037*	
C9	0.7412 (5)	0.2573 (5)	0.2986 (5)	0.0239 (9)	
C10	0.7378 (6)	0.3919 (5)	0.4170 (5)	0.0237 (9)	
C11	0.5237 (7)	0.2779 (6)	−0.0504 (5)	0.0374 (11)	
H11A	0.5022	0.1767	−0.1236	0.056*	
H11B	0.6393	0.3348	−0.0369	0.056*	
H11C	0.4260	0.3153	−0.0856	0.056*	
C12	0.3391 (6)	0.1982 (5)	0.0735 (5)	0.0250 (9)	
C13	0.0293 (6)	0.1986 (6)	0.0410 (6)	0.0375 (11)	
H13A	−0.0441	0.2672	0.0560	0.056*	
H13B	0.0354	0.1623	0.1113	0.056*	
H13C	−0.0256	0.1177	−0.0608	0.056*	
C14	0.6766 (6)	0.6451 (5)	0.4849 (5)	0.0296 (10)	
C15	0.6353 (10)	0.8756 (7)	0.5235 (8)	0.0572 (16)	
H15A	0.6072	0.9287	0.4692	0.086*	
H15B	0.7543	0.9266	0.6029	0.086*	
H15C	0.5434	0.8686	0.5668	0.086*	

### Atomic displacement parameters (Å^2^)

**Table:**

	*U*^11^	*U*^22^	*U*^33^	*U*^12^	*U*^13^	*U*^23^
Br1	0.0640 (4)	0.0637 (5)	0.0362 (3)	0.0183 (3)	0.0110 (3)	0.0345 (3)
O1	0.0334 (17)	0.0248 (17)	0.0434 (19)	0.0079 (14)	0.0182 (14)	0.0139 (15)
O2	0.0225 (15)	0.0373 (18)	0.0417 (18)	0.0120 (13)	0.0114 (13)	0.0257 (15)
O3	0.060 (2)	0.035 (2)	0.0338 (19)	0.0175 (18)	0.0171 (17)	0.0102 (16)
O4	0.054 (2)	0.0290 (18)	0.050 (2)	0.0216 (16)	0.0250 (18)	0.0240 (16)
N1	0.0270 (19)	0.032 (2)	0.0267 (18)	0.0135 (16)	0.0107 (15)	0.0142 (17)
C2	0.024 (2)	0.033 (2)	0.026 (2)	0.0108 (18)	0.0100 (17)	0.0169 (19)
C3	0.022 (2)	0.029 (2)	0.033 (2)	0.0087 (17)	0.0116 (17)	0.0196 (19)
C4	0.024 (2)	0.027 (2)	0.031 (2)	0.0083 (17)	0.0130 (17)	0.0186 (19)
C5	0.033 (2)	0.034 (3)	0.029 (2)	0.010 (2)	0.0124 (18)	0.018 (2)
C6	0.033 (2)	0.041 (3)	0.031 (2)	0.009 (2)	0.0105 (19)	0.025 (2)
C7	0.032 (2)	0.046 (3)	0.048 (3)	0.016 (2)	0.015 (2)	0.035 (3)
C8	0.029 (2)	0.030 (2)	0.036 (2)	0.0144 (19)	0.0129 (19)	0.016 (2)
C9	0.0187 (19)	0.030 (2)	0.029 (2)	0.0077 (17)	0.0115 (16)	0.0171 (18)
C10	0.023 (2)	0.025 (2)	0.028 (2)	0.0072 (17)	0.0108 (16)	0.0157 (18)
C11	0.037 (3)	0.050 (3)	0.032 (2)	0.014 (2)	0.015 (2)	0.024 (2)
C12	0.025 (2)	0.030 (2)	0.0205 (19)	0.0074 (18)	0.0099 (16)	0.0120 (17)
C13	0.022 (2)	0.047 (3)	0.050 (3)	0.007 (2)	0.011 (2)	0.030 (3)
C14	0.026 (2)	0.030 (2)	0.038 (3)	0.0093 (18)	0.0155 (19)	0.019 (2)
C15	0.073 (4)	0.034 (3)	0.079 (4)	0.031 (3)	0.040 (4)	0.029 (3)

### Geometric parameters (Å, º)

**Table:**

Br1—C6	1.905 (5)	C8—H8	0.9300
O2—C13	1.455 (5)	C9—C8	1.395 (6)
O4—C15	1.442 (7)	C10—C5	1.393 (6)
N1—C2	1.452 (6)	C10—C9	1.427 (6)
N1—C9	1.380 (6)	C11—H11A	0.9600
N1—H1	0.899 (10)	C11—H11B	0.9600
C2—C3	1.499 (6)	C11—H11C	0.9600
C2—C11	1.537 (6)	C12—O1	1.197 (6)
C2—C12	1.548 (6)	C12—O2	1.322 (5)
C3—C4	1.344 (6)	C13—H13A	0.9600
C3—H3	0.9300	C13—H13B	0.9600
C4—C10	1.469 (6)	C13—H13C	0.9600
C4—C14	1.493 (6)	C14—O3	1.200 (6)
C5—H5	0.9300	C14—O4	1.344 (6)
C6—C5	1.378 (7)	C15—H15A	0.9600
C7—C6	1.371 (8)	C15—H15B	0.9600
C7—H7	0.9300	C15—H15C	0.9600
C8—C7	1.393 (7)		
			
C12—O2—C13	116.0 (4)	N1—C9—C10	120.0 (4)
C14—O4—C15	114.8 (4)	C8—C9—C10	119.7 (4)
C2—N1—H1	119 (6)	C5—C10—C4	125.1 (4)
C9—N1—C2	120.0 (4)	C5—C10—C9	118.7 (4)
C9—N1—H1	112 (6)	C9—C10—C4	116.2 (4)
N1—C2—C3	109.0 (3)	C2—C11—H11A	109.5
N1—C2—C11	108.4 (4)	C2—C11—H11B	109.5
N1—C2—C12	110.7 (4)	C2—C11—H11C	109.5
C3—C2—C11	112.4 (4)	H11A—C11—H11B	109.5
C3—C2—C12	109.3 (4)	H11A—C11—H11C	109.5
C11—C2—C12	107.0 (3)	H11B—C11—H11C	109.5
C2—C3—H3	118.8	O1—C12—O2	124.9 (4)
C4—C3—C2	122.3 (4)	O1—C12—C2	124.3 (4)
C4—C3—H3	118.8	O2—C12—C2	110.7 (4)
C3—C4—C10	120.3 (4)	O2—C13—H13A	109.5
C3—C4—C14	119.0 (4)	O2—C13—H13B	109.5
C10—C4—C14	120.7 (4)	O2—C13—H13C	109.5
C6—C5—C10	120.0 (4)	H13A—C13—H13B	109.5
C6—C5—H5	120.0	H13A—C13—H13C	109.5
C10—C5—H5	120.0	H13B—C13—H13C	109.5
C5—C6—Br1	119.1 (4)	O3—C14—O4	121.9 (5)
C7—C6—Br1	119.0 (4)	O3—C14—C4	126.2 (4)
C7—C6—C5	122.0 (4)	O4—C14—C4	111.8 (4)
C6—C7—C8	119.5 (4)	O4—C15—H15A	109.5
C6—C7—H7	120.2	O4—C15—H15B	109.5
C8—C7—H7	120.2	O4—C15—H15C	109.5
C7—C8—C9	120.1 (4)	H15A—C15—H15B	109.5
C7—C8—H8	120.0	H15A—C15—H15C	109.5
C9—C8—H8	120.0	H15B—C15—H15C	109.5
N1—C9—C8	120.1 (4)		
			
C9—N1—C2—C3	40.9 (5)	C3—C4—C14—O4	−16.6 (6)
C9—N1—C2—C11	163.5 (4)	C10—C4—C14—O3	−17.1 (7)
C9—N1—C2—C12	−79.4 (5)	C10—C4—C14—O4	165.6 (4)
C2—N1—C9—C8	154.6 (4)	Br1—C6—C5—C10	−179.6 (3)
C2—N1—C9—C10	−29.0 (6)	C7—C6—C5—C10	0.5 (7)
N1—C2—C3—C4	−28.2 (6)	C8—C7—C6—Br1	178.6 (4)
C11—C2—C3—C4	−148.4 (4)	C8—C7—C6—C5	−1.5 (7)
C12—C2—C3—C4	92.9 (5)	C9—C8—C7—C6	1.0 (7)
N1—C2—C12—O1	−17.8 (6)	N1—C9—C8—C7	176.8 (4)
N1—C2—C12—O2	164.7 (3)	C10—C9—C8—C7	0.4 (7)
C3—C2—C12—O1	−137.9 (4)	C4—C10—C5—C6	−177.1 (4)
C3—C2—C12—O2	44.6 (4)	C9—C10—C5—C6	0.9 (6)
C11—C2—C12—O1	100.1 (5)	C4—C10—C9—N1	0.4 (6)
C11—C2—C12—O2	−77.4 (4)	C4—C10—C9—C8	176.9 (4)
C2—C3—C4—C10	3.3 (6)	C5—C10—C9—N1	−177.8 (4)
C2—C3—C4—C14	−174.5 (4)	C5—C10—C9—C8	−1.3 (6)
C3—C4—C10—C5	−169.9 (4)	O1—C12—O2—C13	3.0 (6)
C3—C4—C10—C9	12.0 (6)	C2—C12—O2—C13	−179.5 (4)
C14—C4—C10—C5	7.9 (7)	O3—C14—O4—C15	−2.4 (7)
C14—C4—C10—C9	−170.2 (4)	C4—C14—O4—C15	175.1 (4)
C3—C4—C14—O3	160.7 (5)		

### Hydrogen-bond geometry (Å, º)

**Table:**

*D*—H···*A*	*D*—H	H···*A*	*D*···*A*	*D*—H···*A*
N1—H1···O1^i^	0.90 (8)	2.12 (8)	3.013 (6)	176 (7)

Symmetry code: (i) −*x*+1, −*y*, −*z*.
